# Effect of Neprilysin Inhibition on Left Ventricular Remodeling in Patients With Asymptomatic Left Ventricular Systolic Dysfunction Late After Myocardial Infarction

**DOI:** 10.1161/CIRCULATIONAHA.121.054892

**Published:** 2021-05-13

**Authors:** Kieran F. Docherty, Ross T. Campbell, Katriona J.M. Brooksbank, John G. Dreisbach, Paul Forsyth, Rosemary L. Godeseth, Tracey Hopkins, Alice M. Jackson, Matthew M.Y. Lee, Alex McConnachie, Giles Roditi, Iain B. Squire, Bethany Stanley, Paul Welsh, Pardeep S. Jhund, Mark C. Petrie, John J.V. McMurray

**Affiliations:** 1 Institute of Cardiovascular and Medical Sciences, British Heart Foundation Glasgow Cardiovascular Research Centre (K.F.D., R.T.C., K.J.M.B., R.L.G., T.H., A.M.J., M.M.Y.L., G.R., P.W., P.S.J., M.C.P., J.J.V.M.), University of Glasgow, United Kingdom.; 2 Robertson Centre for Biostatistics, Institute of Health and Wellbeing (A.M., B.S.), University of Glasgow, United Kingdom.; 3 Glasgow Clinical Research Imaging Facility (T.H., G.R.), Queen Elizabeth University Hospital, United Kingdom (R.T.C.).; 4 Golden Jubilee National Hospital, Glasgow, United Kingdom (J.G.D.).; 5 Pharmacy Services, National Health Service Greater Glasgow and Clyde, United Kingdom (P.F.).; 6 Department of Radiology, Glasgow Royal Infirmary, United Kingdom (G.R.).; 7 Department of Cardiovascular Sciences, University of Leicester and National Institute for Health Research Biomedical Research Centre, Glenfield Hospital, United Kingdom (I.B.S.).

**Keywords:** clinical trial, heart failure, myocardial infarction, natriuretic peptides, neprilysin, renin angiotensin aldosterone system

## Abstract

**Background:**

Patients with left ventricular (LV) systolic dysfunction after myocardial infarction are at a high risk of developing heart failure. The addition of neprilysin inhibition to renin angiotensin system inhibition may result in greater attenuation of adverse LV remodeling as a result of increased levels of substrates for neprilysin with vasodilatory, antihypertrophic, antifibrotic, and sympatholytic effects.

**Methods:**

We performed a prospective, multicenter, randomized, double-blind, active-comparator trial comparing sacubitril/valsartan 97/103 mg twice daily with valsartan 160 mg twice daily in patients ≥3 months after myocardial infarction with a LV ejection fraction ≤40% who were taking a renin angiotensin system inhibitor (equivalent dose of ramipril ≥2.5 mg twice daily) and a β-blocker unless contraindicated or intolerant. Patients in New York Heart Association class ≥II or with signs and symptoms of heart failure were excluded. The primary outcome was change from baseline to 52 weeks in LV end-systolic volume index measured using cardiac magnetic resonance imaging. Secondary outcomes included other magnetic resonance imaging measurements of LV remodeling, change in NT-proBNP (N-terminal pro-B-type natriuretic peptide) and high-sensitivity cardiac troponin I, and a patient global assessment of change questionnaire.

**Results:**

From July 2018 to June 2019, we randomized 93 patients with the following characteristics: mean age, 60.7±10.4 years; median time from myocardial infarction, 3.6 years (interquartile range, 1.2–7.2); mean LV ejection fraction, 36.8%±7.1%; and median NT-proBNP, 230 pg/mL (interquartile range, 124–404). Sacubitril/valsartan, compared with valsartan, did not significantly reduce LV end-systolic volume index; adjusted between-group difference, –1.9 mL/m^2^ (95% CI, –4.8 to 1.0); *P*=0.19. There were no significant between-group differences in NT-proBNP, high-sensitivity cardiac troponin I, LV end-diastolic volume index, left atrial volume index, LV ejection fraction, LV mass index, or patient global assessment of change.

**Conclusions:**

In patients with asymptomatic LV systolic dysfunction late after myocardial infarction, treatment with sacubitril/valsartan did not have a significant reverse remodeling effect compared with valsartan.

**Registration:**

URL: https://www.clinicaltrials.gov; Unique identifier: NCT03552575.

## Abstract 2

Supplemental Digital Content is available in the text.

**Clinical Perspective**

**What Is New?**

- Patients with left ventricular systolic dysfunction after myocardial infarction are at high risk of the subsequent development of heart failure with a reduced ejection fraction.
- The addition of a neprilysin inhibitor to a renin angiotensin system inhibitor (sacubitril/valsartan) may reduce this risk by attenuating the process of adverse left ventricular remodeling, which underlies the development of heart failure.
- In this cardiac magnetic resonance imaging trial, in 93 patients with asymptomatic left ventricular systolic dysfunction (left ventricular ejection fraction ≤40%) caused by myocardial infarction (median, 3.6 [interquartile range, 1.2–7.2] years previously), sacubitril/valsartan, compared with valsartan, did not reduce left ventricular volumes or increase left ventricular ejection fraction, and did not reduce NT-proBNP (N-terminal pro-B-type natriuretic peptide) or cardiac troponin I levels.

**What Are the Clinical Implications?**

- Neprilysin inhibition did not have a substantial effect on late left ventricular remodeling in symptomless patients with a left ventricular ejection fraction ≤40% after myocardial infarction.
- Whether neprilysin inhibition has a beneficial effect in more selected patients with greater systolic dysfunction/elevated natriuretic peptides or on early remodeling is worthy of further investigation.

The development of left ventricular (LV) systolic dysfunction (LVSD) as a result of myocardial infarction (MI) increases the subsequent risk of developing heart failure (HF).^1,2^ Progressive dilation of the LV and reduction in stroke volume, a process known as adverse LV remodeling, precedes the development of HF and can occur in the days, weeks, and even years after MI.^3^ Patients can experience a latent asymptomatic period before the development of symptomatic HF despite a significantly reduced LV ejection fraction (LVEF) and dilated LV.^4^ The process of adverse LV remodeling after MI can be attenuated by pharmacological inhibition of the maladaptive neurohumoral system activation that occurs in response to the reduction in stroke volume.^5^ Four different neurohumoral antagonists (angiotensin-converting enzyme [ACE] inhibitors or angiotensin-receptor blockers, β-blockers, and mineralocorticoid-receptor antagonists) have been shown to reduce the risk of developing HF and death in patients at high risk of developing HF after MI, and the benefits of these drugs are, in part, a result of an attenuation of adverse LV remodeling.^2,5–13^

Not all neurohumoral activation after MI (or in HF) is necessarily harmful; the natriuretic peptides, which are secreted by the heart in response to increased wall stress, aim to counteract the adverse effects of activation of the renin angiotensin aldosterone system and sympathetic nervous system by promoting vasodilation, natriuresis, and diuresis, along with inhibiting pathological hypertrophy and fibrosis.^14^ Endogenous levels of the natriuretic peptides can be augmented by inhibition of neprilysin, the enzyme responsible for their breakdown, along with the catabolism of a range of other vasoactive peptides including adrenomedullin, GLP-1 (glucagon-like peptide 1), apelin, and bradykinin.^15^ The addition of a neprilysin inhibitor to an angiotensin II type 1 receptor blocker, in the form of sacubitril/valsartan, has been shown to reduce the risk of cardiovascular death or HF hospitalization in patients with established HF and reduced ejection fraction (HFrEF) when compared with renin angiotensin system (RAS) inhibition alone with the ACE inhibitor enalapril.^16^ Given their vasodilatory, antihypertrophic, antifibrotic, and sympatholytic effects, along with the clinical benefits observed in patients with HFrEF, the augmentation of natriuretic peptides and other substrates for neprilysin with a neprilysin inhibitor is an attractive therapeutic proposal to prevent or delay adverse LV remodeling after MI, thereby reducing the attendant risk of developing HF.

Consequently, we designed a prospective, multicenter, randomized, double-blind, active-comparator trial powered to investigate the effects of the addition of neprilysin inhibition to RAS inhibition on LV volumes in patients with asymptomatic LVSD after MI.

## Methods

The design and methods of the trial have been published.^17^ The trial protocol and any subsequent substantial amendments were approved by the East of Scotland Research Ethics Committee. All patients provided written consent. The trial is registered (URL: http://www.clinicaltrials.gov. Unique identifier: NCT03552575). The data supporting the findings of this study will be available from the corresponding author on reasonable request.

### Patients

Patients age ≥18 years were eligible if they had an LVEF ≤40% as measured by Simpson’s biplane using transthoracic echocardiography at least 3 months after acute MI without any signs or symptoms of HF (ie, New York Heart Association class I), were taking a minimum dose or greater of ACE inhibitors/angiotensin-receptor blockers (ramipril 2.5 mg twice daily or equivalent), or were able to tolerate such a dose, were treated with a β-blocker, unless intolerant or contraindicated, and had a systolic blood pressure ≥100 mm Hg. Patients were ineligible if they had permanent or persistent atrial fibrillation, an estimated glomerular filtration rate of <30 mL/min per 1.73 m^2^, or a serum potassium level of >5.2 mmol/L. Full inclusion and exclusion criteria are detailed in Table I in the Data Supplement.

### Randomization and Stratification

Patients were randomly assigned 1:1 to either sacubitril valsartan (target dose, 97/103 mg twice daily) or valsartan (target dose, 160 mg twice daily) by using an interactive web response system. The randomization sequence was created using randomized permuted blocks, with block lengths of 4 and 6 (at random), and was stratified by baseline LV end-systolic volume index (LVESVI) measured using cardiac magnetic resonance imaging (MRI) (≤45 mL/m^2^ or >45 mL/m^2^) and by use of diuretics at baseline.

### Schedule of Study Visits

Patients attended for 10 visits: screening (week –12 to 0), randomization (week 0), and weeks 1, 2, 4, 5, 14, 26, 39, and 52 (Table II in the Data Supplement). Because of the coronavirus disease 2019 (COVID-19) pandemic, patients scheduled for the 52-week visit after March 23, 2020, had this visit at an earlier (n=4 [earliest=48 weeks]) or later time point (n=16 [latest=62 weeks]). All patients remained on the study drug until the end-of-trial visit.

### Study Drug

Study drug and matched placebo were commenced at 1 of 3 doses (sacubitril/valsartan 24/26 mg, 49/51 mg, and 97/103 mg twice daily or valsartan 40 mg, 80 mg, and 160 mg twice daily) depending on renal function, blood pressure, and ACE inhibitor or angiotensin-receptor blocker dose at randomization. Uptitration to target dose was attempted during the first 4 weeks after randomization depending on the safety criteria detailed in Figure I in the Data Supplement. Regular monitoring of blood pressure and renal function was performed at all visits during follow-up. All patients and trial staff were blinded to treatment allocation.

### Primary Outcome

The primary outcome was the change from baseline to 52 weeks in LVESVI, measured using cardiac MRI, and indexed for body surface area.

### Secondary Outcomes

Secondary outcomes, measured as change from baseline to 52 weeks, were NT-proBNP (N-terminal pro-B-type natriuretic peptide), high-sensitivity cardiac troponin I, LV end-diastolic volume indexed for body surface area (LVEDVI), left atrial volume indexed for body surface area, LVEF, LV mass indexed for body surface area, and change in patient well-being as assessed using a patient global assessment questionnaire.

### Exploratory Outcomes

A range of biomarkers relating to neprilysin inhibition, neurohumoral activation, and cardiac remodeling was measured from blood and urine samples collected at baseline, 26 weeks, and 52 weeks as exploratory outcomes.

### Cardiac MRI Acquisition and Analysis

ECG-gated cardiac MRI was performed at 1 center (Glasgow Clinical Research Imaging Facility, Queen Elizabeth University Hospital) at baseline prerandomization and week 52 using a 3.0 Tesla scanner (MAGNETOM Prisma, Siemens Healthcare). The imaging protocol included balanced steady-state free precession cine imaging, native T1 mapping (modified Look-Locker inversion-recovery), and delayed gadolinium enhancement sequences. Further details about the MRI protocol and analysis are available in the design article.^17^ All scans were reported by 1 European Association of Cardiovascular Imaging cardiac MRI–certified observer blinded to treatment allocation.

### Biomarker Assessment

High-sensitivity cardiac troponin I, B-type natriuretic peptide, and galectin-3 (Architect i1000SR, Abbott Laboratories, Abbott Diagnostics, Abbot Park, IL) were measured, along with NT-proBNP and growth differentiation factor-15 (Cobas e411, Roche Diagnostics, Rotkreuz, Switzerland), as well as midregional proatrial natriuretic peptide and midregional proadrenomedullin (B·R·A·H·M·S KRYPTOR Compact PLUS, Thermo Fisher Diagnostics, Henningsdorf, Germany), on clinical immunoassay platforms using the manufacturers’ calibrators and quality control materials. Urinary cyclic GMP (cGMP), endothelin-1, tissue inhibitor of metallopeptidase-1, matrix metallopeptidase-9 (using platelet poor plasma), and soluble suppression of tumorigenicity-2 were measured using a commercially available ELISA (R&D Systems, Bio-Techne, Minneapolis, MN), and using the manufacturers’ quality control materials. GLP-1 (plasma from a BD p800 protease inhibitor vacutainer, using Total GLP-1 assay, Mercodia, Uppsala, Sweden), α-ANP (α-atrial natriuretic peptide), C-type natriuretic peptide, apelin (aprotinin-treated plasma, α-ANP[1–28], C-type natriuretic peptide-22, and apelin-36 extraction-free enzyme immunoassays, Phoenix Pharmaceuticals, Burlingame, CA), and procollagen III N-terminal peptide (Tecan, IBL International, Männedorf, Switzerland) were also measured using commercial ELISA assays and the manufacturers’ quality control materials.

### Statistical Analysis

Statistical analyses were conducted at the study data center (Clinical Trials Unit, Robertson Center for Biostatistics, University of Glasgow) according to a prespecified Statistical Analysis Plan. All analyses were performed according to the intention-to-treat principle, including all randomly assigned participants with postrandomization data available for the outcome of interest at any given time point, irrespective of their subsequent participation in the study and their adherence to randomized treatment. Trial sample size was 100 patients on the basis of the calculation that 45 patients in each treatment group provided >90% power (α level=0.05) to detect a mean between-group difference in change in LVESVI from baseline of 6 mL/m^2^ (SD of change=7.8 mL/m^2^), accounting for a discontinuation rate of 10% (lost to follow-up, development of HF, or death).^17^ Data were summarized descriptively for each randomized treatment group, using counts and percentages for categorical variables and mean (SD), or median, 25th and 75th percentiles (interquartile range), depending on the distribution of the data. Each outcome was analyzed using a linear regression analysis model adjusted for randomized treatment, the baseline value of the outcome in question, and use of diuretic at baseline. MRI outcomes also included adjustment for the time from baseline to follow-up MRI. The regression model coefficients for the treatment indicators variable are reported as adjusted between-treatment group mean differences for outcomes at 52 weeks. Log transformations were performed where required to satisfy modeling assumptions, with regression coefficients back-transformed, and interpretable as relative differences. Between-treatment group difference in the patient global assessment of change questionnaire was assessed by means of a Fisher exact test. In a post hoc analysis, we examined for the effect of any modification of treatment effect on the primary outcome by baseline NT-proBNP level using a linear regression model with interaction between treatment group and baseline NT-proBNP (examined as a categorical variable below or at and above the median baseline level [230 pg/mL]), adjusted for randomized treatment, baseline LVESVI, use of diuretics at baseline, and time from randomization to cardiac MRI. A *P* value <0.05 was considered statistically significant. All analyses were conducted using R Studio and R version 4.0.0 (R Foundation for Statistical Computing, Vienna, Austria).

## Results

Recruitment took place between July 2018 and June 2019; follow-up visits were completed in June 2020. Of 158 patients screened from 7 sites in the National Health Service Greater Glasgow and Clyde Health Board, 93 were randomly assigned (47 to sacubitril/valsartan and 46 to valsartan).

### Baseline Characteristics

The baseline characteristics of patients summarized by randomized treatment allocation are displayed in Table 1. The mean (SD) age was 60.7 (10.4) years, and 85 patients (91.4%) were male. The median time from MI was 3.6 years (interquartile range, 1.2–7.2). The index MI was an ST-elevation MI in 90 (96.8%) patients and in the anterior location in 88 (94.6%) patients, and most patients (89 [95.7%]) had received percutaneous or surgical revascularization as treatment for the MI. A β-blocker was taken by 87 (93.5%) patients, a mineralocorticoid-receptor antagonist by 40 (43%), and a loop diuretic by 11 (11.8%). The mean (SD) cardiac MRI LVEF was 36.8% (7.1%), and median NT-proBNP was 230 pg/mL (interquartile range, 124–404).

**Table 1.**
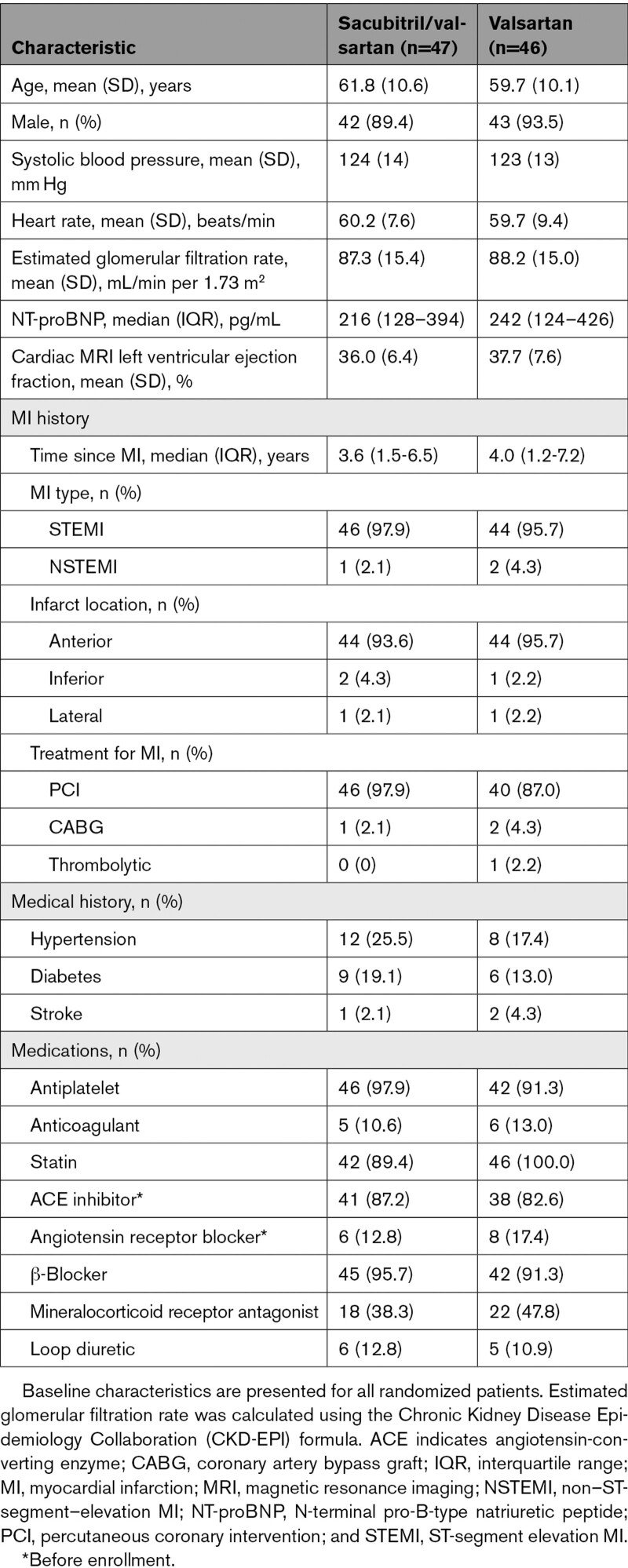
Baseline Characteristics of Randomized Patients

### Completeness of Follow-Up and Adherence

Of the 47 patients randomized to sacubitril/valsartan, 46 remained on randomized therapy and had complete primary outcome data at baseline and week 52 (Figure II in the Data Supplement). Of the 46 patients randomly assigned to valsartan, 46 remained on randomized therapy, and 44 had complete primary outcome data at baseline and week 52. There was 1 death (sudden cardiac death) in the sacubitril/valsartan group, and no deaths in the valsartan group. Among the living patients at the end of the trial, 42 of 46 (91.3%) were taking the target dose of sacubitril/valsartan (97/103 mg twice daily), and 46 of 46 (100%) were taking the target dose of valsartan (160 mg twice daily).

### Primary Outcome

**Figure 1.**
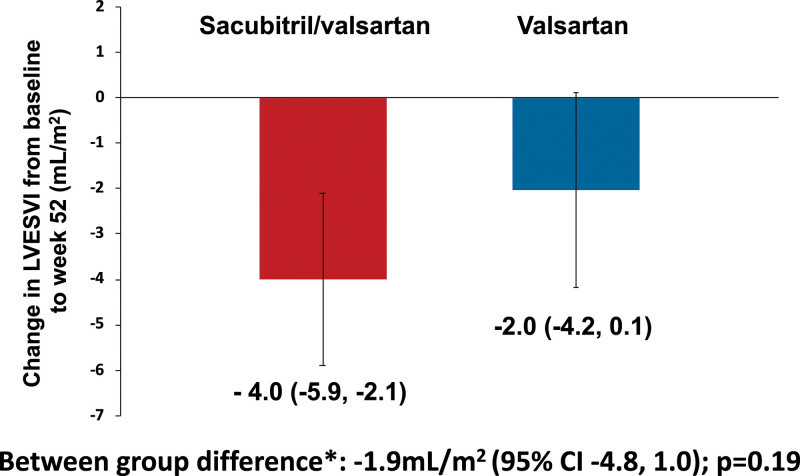
**Change in LVESVI from baseline to week 52.** Data presented as mean and error bars represent 95% CIs. *Calculated using a linear regression model adjusted for randomized treatment, baseline value of the outcome, use of diuretics at baseline, and time from randomization to cardiac magnetic resonance imaging. LVESVI indicates left ventricular end-systolic volume index.

LVESVI decreased by 4.0±6.6 mL/m^2^ between baseline and 52 weeks in the sacubitril/valsartan group and by 2.0±7.3 mL/m^2^ in the valsartan group: adjusted between-group difference, –1.9 (95% CI, –4.8 to 1.0) mL/m^2^; *P*=0.19 (Table 2 and Figure 1). In a post hoc analysis, there was a nominally significant interaction between baseline NT-proBNP and randomized treatment effect (interaction *P* value=0.036). Subgroup analyses of patients below and at or above the median NT-proBNP level at baseline (230 pg/mL) suggested an effect with sacubitril/valsartan in patients at or above the median (adjusted between-group difference, –5.1 mL/m^2^ [95% CI, –9.2 to –1.0]) but not in those below the median (adjusted between-group difference, 1.3 mL/m^2^ [95% CI, –2.9 to 5.5]; Figure III in the Data Supplement).

**Table 2.**
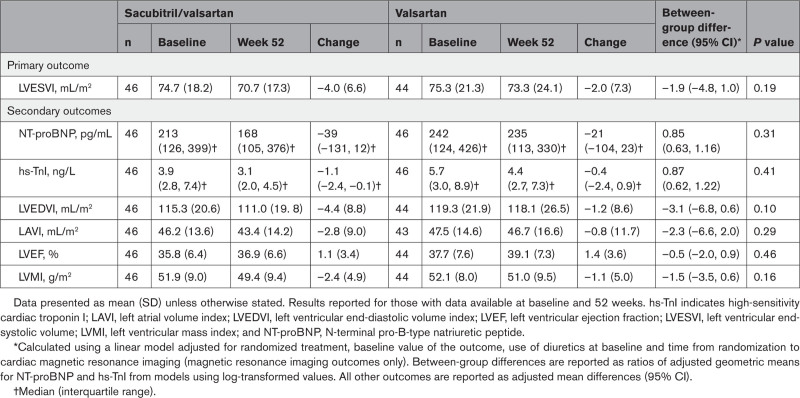
Change in Primary and Secondary Outcomes With Sacubitril/Valsartan or Valsartan From Baseline to Week 52

### Secondary Outcomes

#### NT-proBNP and Troponin

There were no significant between-group differences after 52 weeks of treatment with sacubitril/valsartan or valsartan in either NT-proBNP or high-sensitivity cardiac troponin I (Table 2).

#### Cardiac MRI

**Figure 2.**
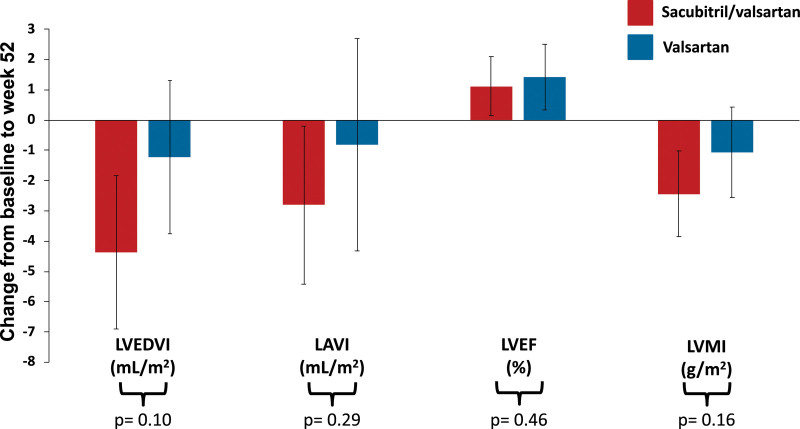
**Change in secondary cardiac MRI outcomes from baseline to week 52.** Data presented as mean and error bars represent 95% CIs. Between-group difference calculated using a linear regression model adjusted for randomized treatment, baseline value of the outcome, use of diuretics at baseline, and time from randomization to cardiac magnetic resonance imaging. LAVI indicates left atrial volume index; LVEDVI, left ventricular end-diastolic volume index; LVEF, left ventricular ejection fraction; and LVMI, left ventricular mass index.

LVEDVI (between-group difference, –3.1 mL/m^2^ [95% CI, –6.8, 0.6]), left atrial volume index (–2.3 mL/m^2^ [95% CI, –6.6, 2.0]), and LV mass index (–1.5 g/m^2^ [95% CI, –3.5, 0.6]) all decreased to a greater degree with sacubitril/valsartan compared with valsartan; however, none of the between-group differences were statistically significant (all *P*≥0.05; Table 2 and Figure 2). There also was no significant between-group difference in LVEF at week 52 (–0.5% [95% CI, –2.0, 0.9]; *P*=0.46; Table 2 and Figure 2).

#### Patient Global Assessment

Data on the patient global assessment were available for 92 patients (46 in both treatment groups) at 52 weeks. An improvement in general well-being from baseline was reported by 22 (47.8%) and 25 (54.3%) in the sacubitril/valsartan and valsartan groups, respectively, with no significant between-group difference (*P*=0.56).

#### Exploratory Biomarker Outcomes

Sacubitril/valsartan, compared with valsartan, increased plasma levels of ANP (*P*=0.013), midregional proadrenomedullin (*P*<0.001), GLP-1 (*P*<0.001), galectin-3 (*P*=0.045), and urinary cGMP (*P*=0.001). Midregional proatrial natriuretic peptide was significantly reduced with sacubitril/valsartan (*P*=0.009). There were no other significant between-group differences in other biomarkers (Table 3).

**Table 3.**
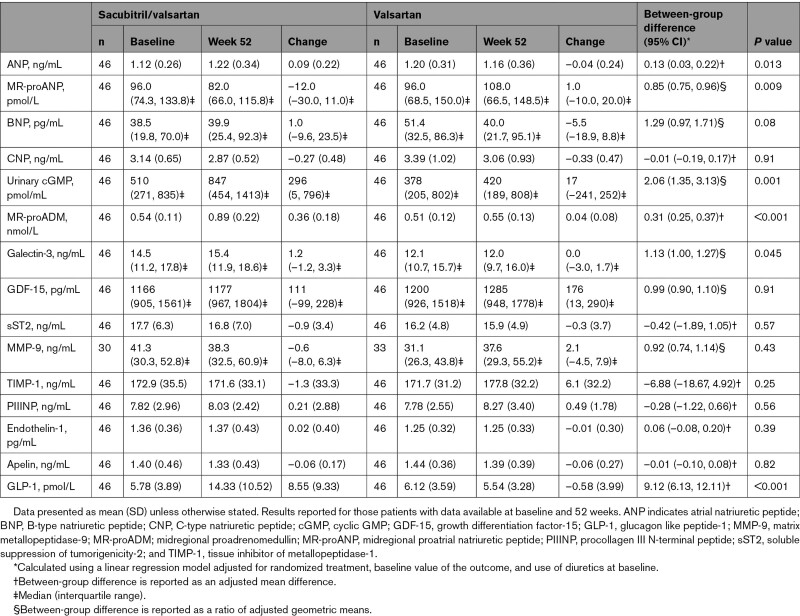
Change in Exploratory Biomarkers With Sacubitril/Valsartan or Valsartan From Baseline to Week 52

#### Safety

Adverse events of interest are summarized by randomized treatment in Table III in the Data Supplement. There were few cases of worsening renal function or hyperkalemia with no significant between-group differences. There were numerically more cases of symptomatic hypotension with sacubitril/valsartan than valsartan (n=7 versus n=1). No cases of symptomatic hypotension required permanent discontinuation of study treatment. Compared with baseline, change in systolic blood pressure at 52 weeks was –5.8 (16.5) mm Hg in the sacubitril/valsartan group and +0.17 (16.8) mm Hg in the valsartan group, yielding a between-group adjusted mean difference of –5.3 mm Hg (95% CI, –11.5 to 1.0); *P*=0.10.

## Discussion

In patients with asymptomatic LVSD as a result of a previous MI, we found that the addition of a neprilysin inhibitor to RAS inhibition with sacubitril/valsartan, compared with RAS inhibition alone with valsartan, did not lead to significant favorable changes in LV or atrial volumes, LVEF, and biomarkers of LV wall stress (NT-proBNP) or myocardial damage (high-sensitivity cardiac troponin I).

The common link between MI, the development of HFrEF, and worsening of established HFrEF is progressive, pathological LV remodeling.^3,18^ The benefits of neurohumoral antagonists in patients at high risk of HF after MI and in those with established HFrEF are, in part, the ability of these drugs to prevent, delay, or even reverse LV remodeling. In patients with HFrEF, the beneficial effects of pharmacological or device therapies on LV volumes and function have been shown to significantly correlate with the therapy’s treatment effect on mortality.^19^ Therefore, given the clinical benefits observed with sacubitril/valsartan in patients with HFrEF in the PARADIGM-HF trial (Prospective Comparison of Angiotensin Receptor–Neprilysin Inhibitor With ACE Inhibitor to Determine Impact on Global Mortality and Morbidity in Heart Failure), it is not unreasonable to hypothesize that these benefits may be a result, in part, of a positive effect on LV remodeling.^16^ Indeed, a series of preclinical models have reported positive effects of neprilysin inhibition on LV volumes and function, along with attenuating fibrosis, 1 of the key processes underlying adverse remodeling.^20,21^ Given the common pathophysiology in patients with asymptomatic LVSD after MI and those with symptomatic HFrEF and the established benefits of neurohumoral antagonists in both groups, it is therefore perhaps surprising that we did not observe a significant reverse remodeling effect with the addition of neprilysin inhibition.

After the results of PARADIGM-HF, data on the remodeling effect of neprilysin inhibition have been examined in 2 randomized controlled trials. In the EVALUATE-HF trial (Effect of Sacubitril-Valsartan versus Enalapril on Aortic Stiffness in Patients With Heart Failure and Reduced Ejection Fraction), sacubitril/valsartan, in comparison with enalapril, did not have a significant effect on the primary end point of central aortic stiffness but significantly reduced the secondary echocardiography end points of LVESVI by 1.6 mL/m^2^, LVEDVI by 2.0 mL/m^2^, and left atrial volume index by 2.8 mL/m^2^, with no difference in LVEF after 12 weeks in patients with HFrEF, the majority of whom were symptomatic (ie, New York Heart Association class ≥II).^22^ The magnitude of these changes was less than those observed with other established HFrEF treatments; however, this may simply reflect the relatively short follow-up time of EVALUATE-HF.^23–26^ In the PRIME trial (Pharmacological Reduction of Functional, Ischemic Mitral Regurgitation), in patients with significant functional mitral regurgitation and LVEF between 25% and <50%, 12 months of treatment with sacubitril/valsartan, compared with valsartan, reduced LVEDVI by 7.0 mL/m^2^ with no effect on LVESVI or LVEF.^27^ An observational study, PROVE-HF (Prospective Study of Biomarkers, Symptom Improvement, and Ventricular Remodeling During Sacubitril/Valsartan Therapy for Heart Failure), reported an association between the degree of reduction in NT-proBNP with sacubitril/valsartan and reverse LV remodeling; however, because of its nonrandomized design, this study is limited in its ability to draw conclusions about treatment effect.^28^

The population enrolled in our trial is distinct from those studied previously in several ways. First, patients in the present trial were asymptomatic of their LVSD, and this is reflected in lower levels of NT-proBNP in our study (median, 230 pg/mL) compared with EVALUATE-HF (575 pg/mL) and in another contemporary trial demonstrating a significant reverse remodeling effect of the sodium-glucose cotransporter 2 inhibitor empagliflozin in patients with symptomatic HFrEF (466 pg/mL).^22,26^ In addition to PROVE-HF, EVALUATE-HF reported a significant correlation between the degree of change in LVESVI and the change in NT-proBNP from baseline; a similar result was observed in the present trial (Pearson ρ=0.38; *P*<0.001). This finding, along with the potential of a treatment-effect interaction with baseline NT-proBNP level, raises the possibility that reversal or attenuation of LV remodeling with sacubitril/valsartan may be requisite on increased LV wall stress (as evidenced by elevated natriuretic peptide levels), the magnitude of which correlates with the level of neurohumoral activation, progressive adverse remodeling, symptoms, and prognosis.^29,30^ Furthermore, if any remodeling effect of neprilysin inhibition is secondary to hemodynamic improvements as a result of increased vasodilation and augmented diuresis (thereby reducing preload and afterload), then it follows that this effect may be attenuated in patients without evidence of increased LV end-diastolic pressure (ie, elevated natriuretic peptides). A benefit of sacubitril/valsartan on LV remodeling might have been demonstrated if patients had been enrolled on the basis of elevated natriuretic peptide levels, although this is a hypothetical proposal on the basis of a small and post hoc subgroup analysis and needs to be tested prospectively.

A differential reverse remodeling effect has previously been reported with other HF pharmacotherapies, with less effect in asymptomatic patients compared with symptomatic patients; in the REVERT trial (Reversal of Ventricular Remodeling with Toprol-XL), along with no significant difference in LVEDVI compared with placebo, the degree of improvement in LVESVI and LVEF with the β-blocker metoprolol succinate was less in an asymptomatic cohort (with low natriuretic peptide levels) than that in studies of symptomatic patients with HFrEF.^24,31,32^ With ivabradine, the reverse remodeling effect was less in patients with stable coronary artery disease and LVSD (88% had a previous MI) than in patients with symptomatic HFrEF of mixed etiology.^25,33^ A similar finding in radionuclide ventriculogram–measured LVEDV was observed with the ACE inhibitor enalapril in asymptomatic patients enrolled in the prevention arm of the SOLVD trial (Studies of Left Ventricular Dysfunction) when compared with the treatment arm, which included symptomatic patients; however, this finding was not replicated in a larger echocardiography substudy.^23,34^ Given the established clinical benefits of sacubitril/valsartan in symptomatic patients with HFrEF, it would have been unethical for us to randomize symptomatic patients in a 52-week-long remodeling trial, thereby limiting our ability to assess the remodeling effect of neprilysin inhibition in symptomatic patients with HFrEF.

It is also worth highlighting that patients in our population were remote from their index myocardial damage, with a median time from MI of 3.6 years. We mandated that patients must be at least 3 months after MI, to minimize the degree of LV stunning and transient systolic dysfunction, which can be seen acutely after MI. It may be, however, that the addition of neprilysin inhibition may be more beneficial in this early time period when there is pronounced neurohumoral activation and before the development of fibrosis and myocardial scar. Indeed, in the short-term trials conducted early after acute MI, most of the mortality benefit with ACE inhibitors was seen in the first week after MI.^35^ Furthermore, in the placebo-controlled SAVE trial (Survival and Ventricular Enlargement), when started in patients with LVSD immediately after MI, the ACE inhibitor captopril was found to attenuate progressive LV dilatation in the first but not the second year after MI.^36^ The potential benefit of neprilysin inhibition in high-risk patients started in the week after MI is being examined in the PARADISE-MI outcome trial (Prospective Angiotensin Receptor–Neprilysin Inhibitor Versus ACE Inhibitor Trial to Determine Superiority in Reducing Heart Failure Events After MI; URL: http://www.clinicaltrials.gov. Unique identifier: NCT02924727), which will report later this year and which includes an echocardiography substudy.^37^

The results of our exploratory biomarker analyses provide some novel insights into the mechanisms of action of neprilysin inhibition. A strength of the present trial was the use of valsartan as the comparator, allowing us to examine the effect of neprilysin per se on both remodeling indices and biomarkers. Consistent with the greater affinity neprilysin has for ANP relative to B-type natriuretic peptide, we observed a significant increase in ANP with sacubitril/valsartan, but the change in B-type natriuretic peptide was not statistically significant.^38^ Along with an increase in urinary cGMP and a significant reduction in midregional proatrial natriuretic peptide (a marker of ANP production and not a substrate for neprilysin), these results suggest that increased ANP bioactivity (secondary to reduced breakdown by neprilysin) through the cGMP pathway may play a key role in the mechanism of action of neprilysin inhibition. We did not detect any difference in levels of C-type natriuretic peptide, perhaps reflecting the relatively low circulating levels of this peptide.^39^ Adrenomedullin is a potent vasodilator as well as having positive inotropic, antifibrotic, and natriuretic effects. We observed a significant increase in midregional proadrenomedullin, a marker of the precursor of bioactive adrenomedullin (ie, not a substrate for neprilysin). This result could represent a degree of assay cross-reactivity with bioactive adrenomedullin (a substrate for neprilysin) or could support an upregulation of adrenomedullin production secondary to the observed increase in ANP.^40^ Conversely, it has also been demonstrated that administration of adrenomedullin augments natriuretic peptide levels.^41,42^ Taken together, these results suggest an interaction between the activity of these peptides. Nevertheless, an increase in both peptides is thought to be favorable. Last, we observed a significant increase in GLP-1, an incretin hormone, which also has beneficial cardiovascular effects including improved cardiac glucose utilization, natriuresis, myocardial function, and vasodilation.^43^ Inhibition of dipeptidyl peptidase-4, the enzyme that, along with neprilysin, breaks down GLP-1, did not improve outcomes in patients with type 2 diabetes after MI.^44^ Furthermore, pharmacological agonists of the GLP-1 receptor have not been shown to have beneficial effects on cardiac structure and function in HFrEF; however, this may reflect that unlike native GLP-1, these compounds are not metabolized by dipeptidyl peptidase-4 to GLP-1(9-36), a substrate for neprilysin, the cardioprotective effects of which may be partially independent of its activation of the GLP-1 receptor.^45–47^ We did not observe beneficial effects of the addition of neprilysin inhibition on markers of profibrotic processes, as have previously been reported in PARADIGM-HF; however, the relatively small sample size may have limited our ability to detect small differences.^48^

### Strengths and Limitations

The strengths of the present study include the use of cardiac MRI, the gold standard method of assessing LV volumes and function; the higher than expected ascertainment of primary outcome data; and near complete biomarker results for the cohort.

We only recruited patients with LVSD as a result of a previous MI; it is possible that any reverse remodeling effect is attenuated in patients with ischemic cardiomyopathy compared with those with nonischemic causes, as is seen with cardiac resynchronization therapy.^49^ We deliberately examined the effect of sacubitril/valsartan on late LV remodeling after MI, recruiting patients at least 3 months after an acute event. However, we cannot draw any conclusions about the potential effect of neprilysin inhibition on the early and distinctive remodeling in the acute phase of MI. We did not include patients with atrial fibrillation. Patients only received treatment for 52 weeks, and a longer time period may be required to see a significant beneficial effect in this patient population. Our trial was sized to provide sufficient power to detect a mean between-group difference in LVESVI of 6 mL/m^2^ at 52 weeks. The hazard ratios and 95% CIs for the effect of treatment did not preclude a smaller treatment difference, but the modest prespecified sample size limited our ability to detect such a difference, if it existed. In the PARADIGM-HF trial, sacubitril/valsartan was superior to the ACE inhibitor enalapril in patients with symptomatic HFrEF.^16^ Our findings are not directly comparable given the differences in both the patients studied and comparator therapy. It is also possible that a comparison of sacubitril/valsartan with an ACE inhibitor may show different results in terms of remodeling outcomes than those presented; however, it should be noted that our choice of comparator, the angiotensin-receptor blocker valsartan, was shown to be equivalent to the ACE inhibitor captopril in both improving clinical outcomes and attenuating adverse LV remodeling in high-risk patients after MI.^2,11^ Our biomarker tests are post hoc, and the number of statistical tests raises the possibility of chance findings; we do note, however, that the results for cGMP, midregional proadrenomedullin, and GLP-1 remain significant at a Bonferroni correction *P* value <0.003 (*P*=0.05/15).

## Conclusions

In patients with asymptomatic LVSD late after MI, the addition of a neprilysin inhibitor to standard therapy with a RAS inhibitor and β-blocker did not have a significant reverse remodeling effect or improve biomarkers associated with LV wall stress or myocardial damage.

## Acknowledgments

The authors are grateful to Sister Barbara Meyer, the staff of the National Health Service Greater Glasgow and Clyde Research and Innovation Department, Clinical Research Facility, and the Glasgow Clinical Research Imaging Facility, Queen Elizabeth University Hospital, for their assistance in the setup and running of the trial. Biomarker analyses were performed by Philip Stewart, Elaine Butler, Josephine Cooney, and Emma Dunning at the Glasgow Biomarker Laboratory, Institute of Cardiovascular and Medical Sciences, University of Glasgow.

## Sources of Funding

This trial was funded by the British Heart Foundation (PG/17/23/32850). Trial medication and funding for trial drug packaging, labeling, distribution, storage, and destruction were supplied by Novartis Pharmaceuticals UK Limited, who had no role in the design and conduct of the study; collection, management, analysis, and interpretation of the data; preparation of the article; or decision to submit the article for publication. J.J.V.M. and M.C.P. are supported by a British Heart Foundation Center of Research Excellence Grant (RE/18/6/34217).

## Disclosures

K.F.D. reports personal fees from AstraZeneca and Eli Lilly outside the submitted work. P.F. reports personal fees from Novartis for lectures and scientific advice outside the submitted work. M.M.Y.L.’s employer, the University of Glasgow, has received grant support from Boehringer Ingelheim outside the submitted work. A.M. and B.S. report grants from British Heart Foundation during the conduct of the study. I.B.S. reports grants and personal fees from Novartis outside the submitted work; research grants from Boehringer Ingelheim, Merck, Vifor, and AstraZeneca. P.W. reports receiving grants from Roche Diagnostics, AstraZeneca, and; research grants from Boehringer Ingelheim, Merck, Vifor, and AstraZeneca. Boehringer Ingelheim outside the submitted work. P.S.J. reports personal fees from Novartis, and payments made to his employer (the University of Glasgow) for work on the PARADIGM-HF and PARAGON-HF trials (Prospective Comparison of ARNI With ARB Global Outcomes in HF With Preserved Ejection Fraction) during the conduct of the study; personal fees from AstraZeneca; and grants from Boehringer Ingelheim outside the submitted work. M.C.P. reports receiving grants and personal fees from Novartis; lecture fees during the conduct of the study and personal fees from Novo Nordisk, AstraZeneca, Eli Lilly, Napp Pharmaceuticals, Takeda Pharmaceutical, Alnylam, Bayer, Resverlogix, and Cardiorentis; and grants and personal fees from Boehringer Ingelheim outside the submitted work. J.J.V.M.’s employer, Glasgow University, has been paid by Novartis (who manufactures sacubitril/valsartan) for his time spent as committee member for the trials listed (using sacubitril/valsartan), meetings related to these trials, and other activities related to sacubitril/valsartan, eg, lectures, advisory boards, and other meetings. Novartis has also paid for his travel and accommodations for some of these meetings. These payments were made through a consultancy with Glasgow University, and he has not received personal payments in relation to these trials/this drug. The trials include PARADIGM-HF: co-PI; PARAGON-HF: co-PI; PERSPECTIVE (Prospective Evaluation of Cognitive Function in Heart Failure: Efficacy and Safety of Entresto Compared to Valsartan on Cognitive Function in Patients With Chronic Heart Failure and Preserved Ejection Fraction), PARADISE-MI, and UK HARP III Trial (UK Heart and Renal Protection-III): executive/steering committees. No other conflicts of interest were declared. The other authors report no conflicts.

## Supplemental Materials

Data Supplement Tables I–III

Data Supplement Figures I–III

## Supplementary Material


