# Supplementary material for: Effect of Neprilysin Inhibition on Left Ventricular Remodeling in Patients With Asymptomatic Left Ventricular Systolic Dysfunction Late After Myocardial Infarction
Source: Circulation. 2021 May 13;144(3):199–209. doi: 10.1161/CIRCULATIONAHA.121.054892 (PMC8284373; doi:10.1161/CIRCULATIONAHA.121.054892)
Supplement: Supplementary file 1 [file cir-144-199-s001.pdf]

The effect of neprilysin inhibition on left ventricular remodeling in patients with asymptomatic left ventricular systolic dysfunction late after myocardial infarction

Supplemental material

Supplement Table I: Trial inclusion and exclusion criteria

Inclusion criteria	• Acute myocardial infarction ≥3 months prior to randomisation<br>• Left ventricular ejection ≤40% as measured by transthoracic echocardiography<br>• Ability to provide written, informed consent<br>• Age ≥18 years<br>• Tolerance of a minimum dose of ACE inhibitor/ARB (ramipril 2.5mg b.d. or equivalent)<br>• Treatment with a beta-blocker unless not tolerated or contraindicated.
Exclusion criteria	• Contraindication to cardiac MRI (ferrous prosthesis, implantable cardiac device or severe claustrophobia)<br>• Clinical (NYHA functional class ≥II) and/or radiological heart failure<br>• Symptomatic hypotension and/or systolic blood pressure <100 mmHg<br>• eGFR < 30 mL/min/1.73m^2 and/or serum potassium >5.2mmol/L<br>• Persistent/permanent atrial fibrillation<br>• History of acute myocardial infarction within last 3 months<br>• History of hypersensitivity or allergy to ACE inhibitors/ARB<br>• History of angioedema<br>• Known hypersensitivity to the active study drug substances, contrast media or any of the excipients<br>• Obesity (where body girth exceeds MRI scanner diameter)<br>• Pregnancy, planning pregnancy, or breast feeding<br>• Inability to give informed consent or comply with study protocol

	• Evidence of hepatic disease as determined by any one of the following: AST or ALT values exceeding 2 x ULN at Visit 1, history of hepatic encephalopathy, history of oesophageal varices, or history of portacaval shunt<br>• History of biliary cirrhosis and cholestasis<br>• Active treatment with cholestyramine or colestipol resins<br>• Active treatment with lithium or direct renin inhibitor<br>• Participation in another intervention study involving a drug or device within the past 90 days (co-enrolment in observational studies is permitted)

Abbreviations: ACE, angiotensin converting enzyme; ALT, alanine aminotransferase; ARB, angiotensin receptor blocker; AST, aspartate aminotransferase; NYHA, New York Heart Association; eGFR, estimated glomerular filtration rate; MRI, magnetic resonance imaging; ULN, upper limit of normal.

Supplement Table II: Schedule of Assessments

**Study Procedure**	**Screening (>3months post myocardial infarction)**	**Visit 1 - Randomisation (Week 0 [<3 months following screening])**	**Visit 2 (Week 1 ± 3 days)**	**Visit 3 (Week 2± 3 days)**	**Visit 4 (Week 4± 3 days)**	**Visit 5 (Week 5± 3 days)**	**Visit 6 (Week 14 ± 7 days)**	**Visit 7 (Week 26 ± 7 days)**	**Visit 8 (Week 39 ± 7 days)**	**Visit 9 (Week 52 ± 3 months)**
Review Inclusion/Exclusion Criteria	X	X								
Echocardiogram	X									
Obtain informed consent	X	X								
Cardiac MRI		X								X
Physical examination		X	X		X			X		X
Medical history	X	X								
Concomitant medications	X	X	X	X	X	X	X	X	X	X
Vital Signs (Blood pressure/Heart rate)	X	X	X	X	X	X	X	X	X	X
12 Lead ECG		X								
Spot urine collection		X						X		X
Venepuncture (Urea and Electrolytes/FBC and LFTs)	X[&]	X	X	X	X	X	X	X	X	X
Venepuncture (Biochemical/hormonal/ biomarker analysis)		X						X		X

Pregnancy testing in WoCBP		X			X		X	X	X	X
Patient global assessment questionnaire										X
IMP Dispensing*		X			X		X	X		
Up-titrate IMP#					X					
IMP Administration		X	X	X	X	X	X	X	X	X
Adverse event reporting			X	X	X	X	X	X	X	X
Study completion										X

[*] Study drug will be introduced at equivalent dose to existing ACE inhibitor/ARB treatment (Dose level 2 or 3) at investigator’s discretion
[#]Up-titration not required if patient already on dose level 3
[&]If not done in the preceding 6 months
Abbreviations: MRI, magnetic resonance imaging; ECG, electrocardiogram; FBC, full blood count; LFTs, liver function tests; WoCBP, women of child-bearing potential; IMP, investigational medicinal product.

Supplement Table III: Adverse Events of Interest

**Adverse event**	**Sacubitril/valsartan (n=47)**	**Valsartan (n=46)**
Serum creatinine ≥2.5mg/dl	0	0
Serum potassium >5.5mmol/L	2 (4)	1 (2)
Serum potassium >6.0mmol/L	0	0
Symptomatic hypotension	7 (15)	1 (2)
Symptomatic hypotension with systolic blood pressure <90mmHg	1 (2)	0
Angioedema	0	0
Cough	0	0

n= number of patients with event (%)

Due to the small number of events no tests for statistical significance were performed.

Supplement Figure I: Trial drug initiation and uptitration

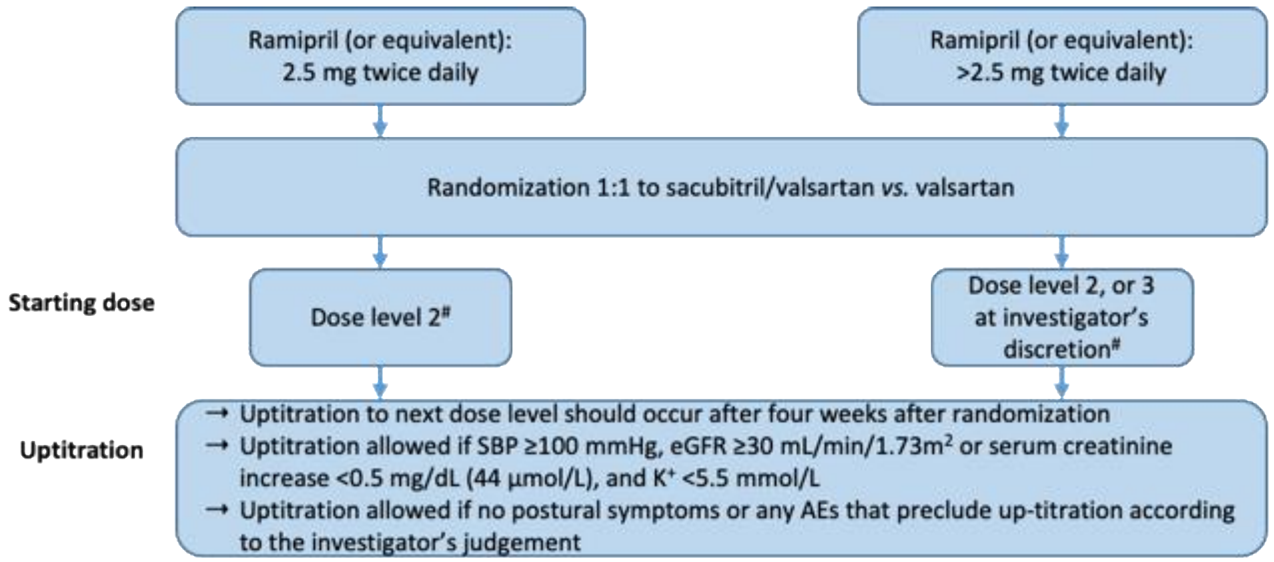

Dose Level	Sacubitril/valsartan arm	Valsartan arm
1	24/26 mg twice daily	40 mg twice daily
2	49/51 mg twice daily	80 mg twice daily
3	97/103 mg twice daily	160 mg twice daily

Dose Level 1 could be considered for patients with systolic blood pressure (SBP) ≥100 to 110 mmHg and/or moderate renal impairment [estimated glomerular filtration rate (eGFR) of 30–60 mL/min/1.73 m^2] at time of randomization.

Abbreviations: AE, adverse event; eGFR, estimated glomerular filtration rate; SBP, systolic blood pressure.

Supplement Figure II: Consort diagram

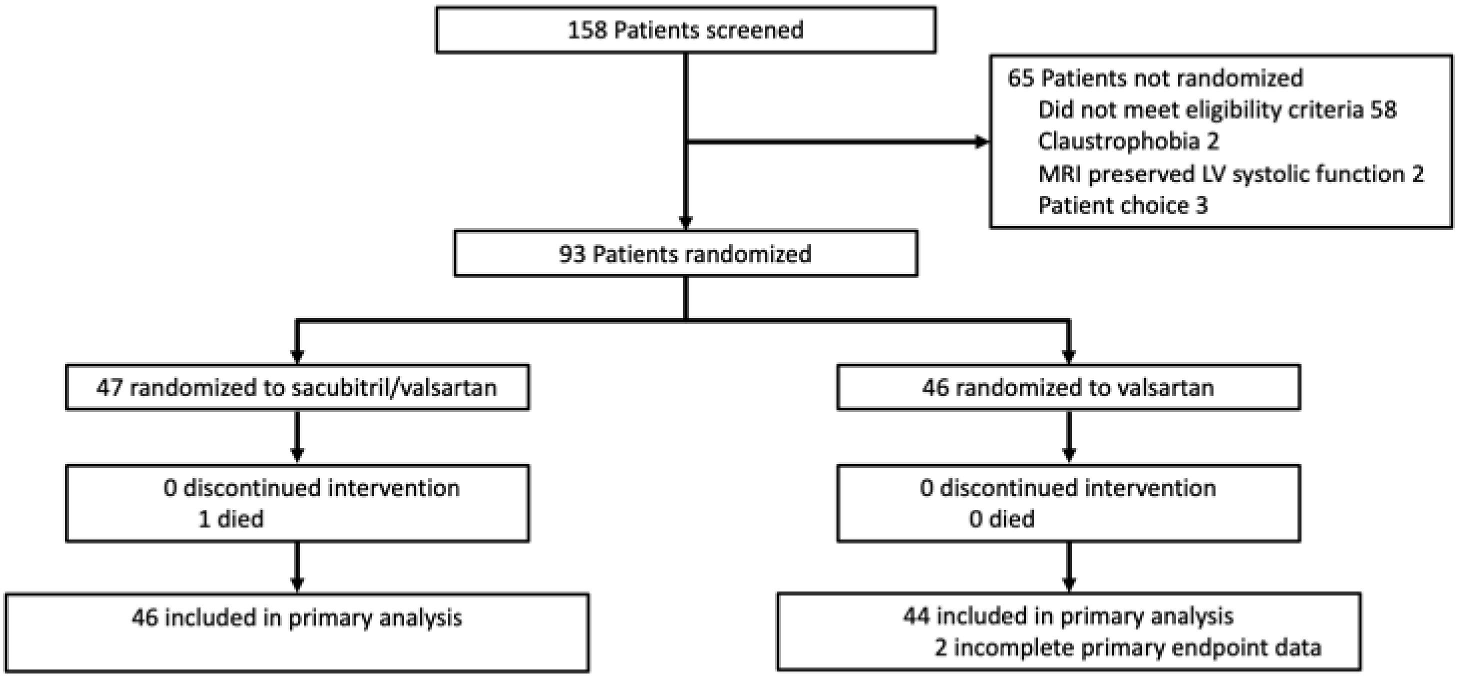

Supplement Figure III: Change in LV end-systolic volume index by baseline NT-proBNP level

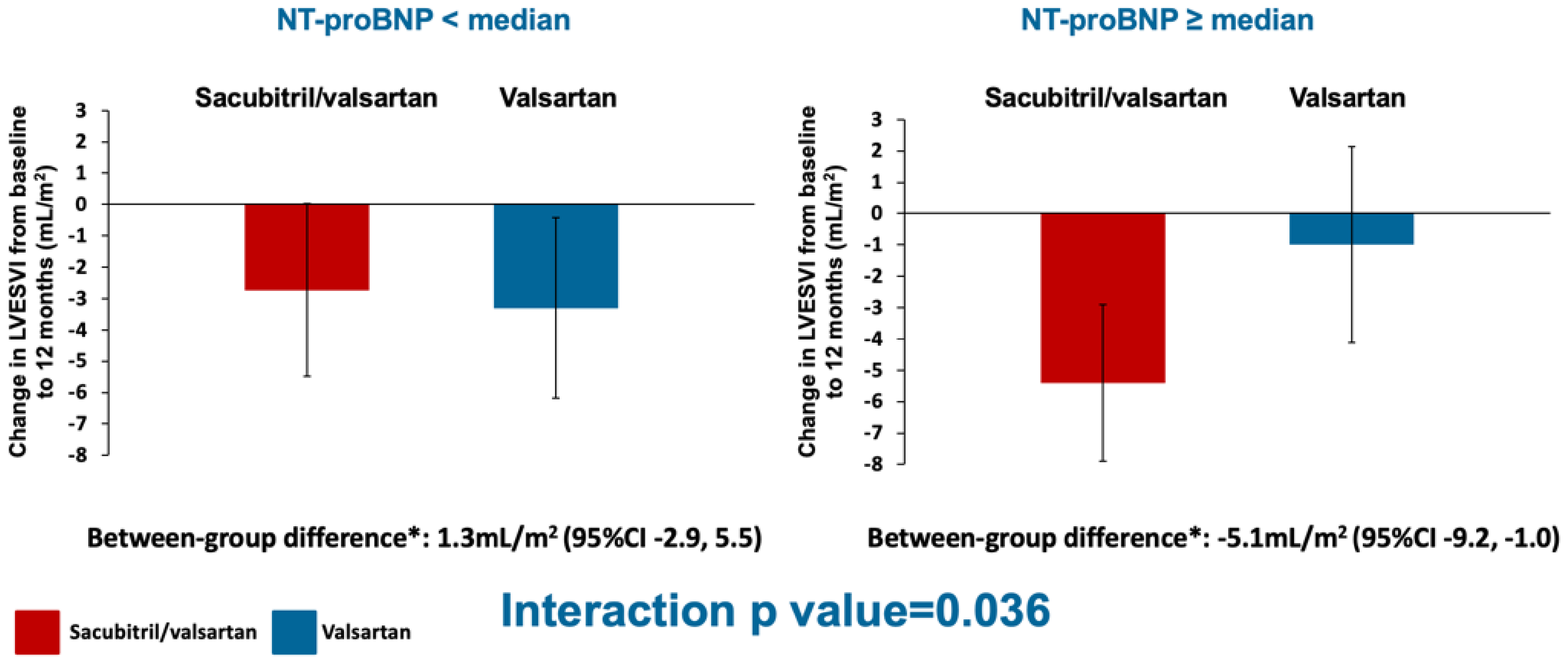

Data presented as mean and error bars represent 95% confidence intervals.
This analysis was *post-hoc.*
Median NT-proBNP was 230 pg/mL
LVESVI, left ventricular end-systolic volume index.
* Treatment effect calculated using a linear regression model with interaction between treatment group and baseline NT-proBNP, adjusted for randomized treatment, baseline value of the outcome, use of diuretics at baseline and time from randomization to cardiac MRI.
